# Supplementary material for: Insulin-like peptides and the mTOR-TFEB pathway protect Caenorhabditis elegans hermaphrodites from mating-induced death
Source: eLife. 2019 Jul 8;8:e46413. doi: 10.7554/eLife.46413 (PMC6697448; doi:10.7554/eLife.46413)
Supplement: Supplementary file 1. — Summary of all the lifespan results in this study. [file elife-46413-supp1.docx]

**Supplementary file 1. Lifespan Analysis Summary**

| **Genotype/condition** | **Mean LS±std.error** | **% change** | **p value** | **N** | **Figure** |
| --- | --- | --- | --- | --- | --- |
| **Experiment 1** |  |  |  |  |  |
| *fog-2(q71)* unmated | 19.7±0.7 | -- | -- | 109 | Fig 1C |
| *fog-2(q71)* Day 3 2h mated with *him-5* males | 15.4±0.7 | -22% | <0.0001 | 78 | Fig 1C |
|  |  |  |  |  |  |
| *fog-2(q71)* unmated | 21.0±0.6 | -- | -- | 107 | Fig 1C |
| *fog-2(q71)* Day 7 2h mated with *him-5* males | 14.8±0.5 | -30% | <0.0001 | 73 | Fig 1C |
|  |  |  |  |  |  |
| **Experiment 2** |  |  |  |  |  |
| *fog-2(q71)* unmated | 20.1±0.6 | -- | -- | 108 |  |
| *fog-2(q71)* Day 3 2h mated with *him-5* males | 15.1±0.8 | -25% | 0.0001 | 82 |  |
|  |  |  |  |  |  |
| *fog-2(q71)* unmated | 18.3±0.7 | -- | -- | 105 |  |
| *fog-2(q71)* Day 7 2h mated with *him-5* males | 14.9±0.6 | -19% | 0.0004 | 89 |  |
|  |  |  |  |  |  |
| **Experiment 3** |  |  |  |  |  |
| *fog-2(q71)* unmated | 18.2±0.8 | -- | -- | 108 | Fig 1D |
| *fog-2(q71)* Day 3 2h mated with *fog-2* males | 12.6±0.7 | -31% | <0.0001 | 92 | Fig 1D |
| *fog-2(q71)* Day 3 2h mated with *fer-6* males | 14.1±0.7 | -23% | 0.0002 | 113 | Fig 1D |
|  |  |  |  |  |  |
| **Experiment 4** |  |  |  |  |  |
| *fog-2(q71)* unmated | 16.4±0.9 | -- | -- | 36 |  |
| *fog-2(q71)* Day 3 2h mated with *fog-2* males | 13.8±0.5 | -16% | 0.0037 | 54 |  |
| *fog-2(q71)* Day 3 2h mated with *fer-6* males | 13.5±0.5 | -18% | 0.0023 | 52 |  |
|  |  |  |  |  |  |
| **Experiment 5** |  |  |  |  |  |
| *fog-2(q71)* unmated | 20.3±1.0 | -- | -- | 50 | Fig 1E |
| *fog-2(q71)* Day 7 2h mated with *fog-2* males | 14.6±0.9 | -28% | <0.0001 | 41 | Fig 1E |
| *fog-2(q71)* Day 7 2h mated with *fer-6* males | 15.6±1.0 | -23% | 0.0016 | 40 | Fig 1E |
|  |  |  |  |  |  |
| **Experiment 6** |  |  |  |  |  |
| *fog-2(q71)* unmated | 16.3±0.6 | -- | -- | 33 |  |
| *fog-2(q71)* Day 7 2h mated with *fog-2* males | 14.3±0.5 | -12% | 0.0023 | 34 |  |
| *fog-2(q71)* Day 7 2h mated with *fer-6* males | 15.2±0.5 | -7% | 0.0384 | 33 |  |
|  |  |  |  |  |  |
| **Experiment 7** |  |  |  |  |  |
| N2 unmated | 11.9±0.6 | -- | -- | 47 | Fig 1F |
| N2 Day 3 2h mated with *daf-22* males | 11.1±0.4 | -7% | 0.3058 | 54 | Fig 1F |
| N2 unmated Day 3 2h on *fog-2* males conditioned plates | 11.7±0.5 | -2% | 0.9696 | 47 | Fig 1F |
|  |  |  |  |  |  |
| **Experiment 8** |  |  |  |  |  |
| N2 unmated | 15.0±0.7 | -- | -- | 47 |  |
| N2 Day 3 2h mated with *daf-22* males | 15.2±0.8 | 1% | 0.6442 | 46 |  |
| N2 unmated Day 3 2h on *fog-2* males conditioned plates | 16.2±0.8 | 8% | 0.1755 | 46 |  |
|  |  |  |  |  |  |
| **Experiment 9** |  |  |  |  |  |
| N2 unmated | 14.3±0.7 | -- | -- | 33 | Fig 1G |
| N2 Day 7 2h mated with *daf-22* males | 11.4±0.4 | -20% | 0.0001 | 45 | Fig 1G |
| N2 unmated Day 7 2h on *fog-2* males conditioned plates | 13.7±0.7 | -4% | 0.5984 | 40 | Fig 1G |
|  |  |  |  |  |  |
| **Experiment 10** |  |  |  |  |  |
| N2 unmated | 15.4±0.7 | -- | -- | 42 |  |
| N2 Day 7 2h mated with *daf-22* males | 11.0±0.5 | -29% | <0.0001 | 40 |  |
| N2 unmated Day 7 2h on *fog-2* males conditioned plates | 15.1±0.9 | -2% | 0.9201 | 33 |  |
|  |  |  |  |  |  |
| **Experiment 11** |  |  |  |  |  |
| N2 unmated | 14.1±0.7 | -- | -- | 36 | Fig 1B |
| N2 Day 3 2h mated with *fog-2* males | 13.9±0.6 | -1% | 0.8481 | 50 | Fig 1B |
|  |  |  |  |  |  |
| N2 unmated | 13.8±0.5 | -- | -- | 53 | Fig 1B |
| N2 Day 7 2h mated with *fog-2* males | 10.0±0.4 | -28% | <0.0001 | 54 | Fig 1B |
|  |  |  |  |  |  |
| **Experiment 12** |  |  |  |  |  |
| *fog-2(q71)* unmated | 15.3±0.8 | -- | -- | 45 | Fig 1H |
| *fog-2(q71)* Day 3 2h mated with *daf-22* males | 10.4±0.6 | -32% | <0.0001 | 54 | Fig 1H |
| *fog-2(q71)* unmated Day 3 2h on *fog-2* males conditioned plates | 16.5±0.8 | +8% | 0.3459 | 48 | Fig 1H |
|  |  |  |  |  |  |
| **Experiment 13** |  |  |  |  |  |
| *fog-2(q71)* unmated | 17.9±0.8 | -- | -- | 60 |  |
| *fog-2(q71)* Day 3 2h mated with *daf-22* males | 13.1±0.5 | -27% | <0.0001 | 62 |  |
| *fog-2(q71)* unmated Day 3 2h on *fog-2* males conditioned plates | 17.6±0.9 | -2% | 0.7848 | 52 |  |
|  |  |  |  |  |  |
| **Experiment 14** |  |  |  |  |  |
| *fog-2(q71)* unmated | 15.5±0.6 | -- | -- | 50 | Fig 1I |
| *fog-2(q71)* Day 7 2h mated with *daf-22* males | 13.1±0.4 | -15% | 0.0017 | 55 | Fig 1I |
| *fog-2(q71)* unmated Day 7 2h on *fog-2* males conditioned plates | 14.7±0.6 | -5% | 0.4493 | 50 | Fig 1I |
|  |  |  |  |  |  |
| **Experiment 15** |  |  |  |  |  |
| *fog-2(q71)* unmated | 20.5±1.0 | -- | -- | 50 |  |
| *fog-2(q71)* Day 7 2h mated with *daf-22* males | 15.6±0.5 | -24% | <0.0001 | 61 |  |
| *fog-2(q71)* unmated Day 7 2h on *fog-2* males conditioned plates | 20.6±1.2 | 0% | 0.9607 | 51 |  |
|  |  |  |  |  |  |
| **Experiment 16** |  |  |  |  |  |
| *fog-2(q71)* unmated | 16.3±0.7 | -- | -- | 58 | Fig 1-S1A |
| *fog-2(q71)* Day 3 2h mated with *fog-2* males | 13.4±0.5 | -18% | 0.0010 | 60 | Fig 1-S1A |
|  |  |  |  |  |  |
| **Experiment 17** |  |  |  |  |  |
| *fem-1(hc17)* unmated | 14.2±0.4 | -- | -- | 75 | Fig 1-S1A |
| *fem-1(hc17)* Day 3 2h mated with *fog-2* males | 11.7±0.3 | -18% | <0.0001 | 80 | Fig 1-S1A |
|  |  |  |  |  |  |
| **Experiment 18** |  |  |  |  |  |
| *daf-16(mu86)* unmated | 12.2±0.5 | -- | -- | 48 | Fig 2A |
| *daf-16(mu86)* Day 3 2h mated with *fog-2* males | 9.5±0.5 | -22% | 0.0003 | 48 | Fig 2A |
|  |  |  |  |  |  |
| N2 unmated | 13.4±0.5 | -- | -- | 61 |  |
| N2 Day 3 2h mated with *fog-2* males | 12.7±0.4 | -5% | 0.2263 | 76 |  |
|  |  |  |  |  |  |
| **Experiment 19** |  |  |  |  |  |
| *daf-16(mu86)* unmated | 11.5±0.3 | -- | -- | 50 |  |
| *daf-16(mu86)* Day 3 2h mated with *fog-2* males | 9.9±0.4 | -14% | 0.0067 | 52 |  |
|  |  |  |  |  |  |
| N2 unmated | 12.7±0.4 | -- | -- | 60 |  |
| N2 Day 3 2h mated with *fog-2* males | 13.3±0.6 | 5% | 0.2742 | 55 |  |
|  |  |  |  |  |  |
| **Experiment 20** |  |  |  |  |  |
| *daf-2(e1370)* unmated | 33.2±2.6 | -- | -- | 39 | Fig 2B |
| *daf-2(e1370)* Day 8 2h mated with *fog-2* males | 32.7±2.9 | -2% | 0.7519 | 39 | Fig 2B |
|  |  |  |  |  |  |
| N2 unmated | 15.4±0.7 | -- | -- | 42 |  |
| N2 Day 7 2h mated with *daf-22* males | 10.8±0.6 | -30% | <0.0001 | 45 |  |
|  |  |  |  |  |  |
| **Experiment 21** |  |  |  |  |  |
| *daf-2(e1370)* unmated | 33.0±2.1 | -- | -- | 40 |  |
| *daf-2(e1370)* Day 8 2h mated with *fog-2* males | 31.8±1.9 | -4% | 0.6772 | 50 |  |
|  |  |  |  |  |  |
| **Experiment 22** |  |  |  |  |  |
| N2 on *ins-37* RNAi unmated | 13.6±0.4 | -- | -- | 77 | Fig 3C |
| N2 on *ins-37* RNAi Day 3 2h mated with *fog-2* males | 12.0±0.4 | -12% | 0.0040 | 66 | Fig 3C |
|  |  |  |  |  |  |
| N2 on l4440 control RNAi unmated | 12.1±0.5 | -- | -- | 66 | Fig 3-S2B |
| N2 on l4440 control RNAi Day 3 2h mated with *fog-2* males | 11.6±0.5 | -4% | 0.6335 | 64 | Fig 3-S2B |
|  |  |  |  |  |  |
| **Experiment 23** |  |  |  |  |  |
| N2 on *ins-37* RNAi unmated | 12.1±0.4 | -- | -- | 55 |  |
| N2 on *ins-37* RNAi Day 3 2h mated with *fog-2* males | 10.4±0.3 | -14% | 0.0006 | 50 |  |
|  |  |  |  |  |  |
| N2 on l4440 control RNAi unmated | 14.9±0.3 | -- | -- | 55 |  |
| N2 on l4440 control RNAi Day 3 2h mated with *fog-2* males | 14.9±0.4 | 0% | 0.7888 | 49 |  |
|  |  |  |  |  |  |
| **Experiment 24** |  |  |  |  |  |
| N2 on *ins-37* RNAi unmated | 14.7±0.5 | -- | -- | 62 |  |
| N2 on *ins-37* RNAi Day 3 2h mated with *fog-2* males | 12.2±0.4 | -17% | 0.0001 | 43 |  |
|  |  |  |  |  |  |
| N2 on l4440 control RNAi unmated | 13.0±0.4 | -- | -- | 50 |  |
| N2 on l4440 control RNAi Day 3 2h mated with *fog-2* males | 12.8±0.4 | -2% | 0.8824 | 50 |  |
|  |  |  |  |  |  |
| **Experiment 25** |  |  |  |  |  |
| *hlh-30(tm1978)* unmated | 13.5±0.5 | -- | -- | 48 | Fig 4A |
| *hlh-30(tm1978)* Day 3 2h mated with *fog-2* males | 10.7±0.4 | -21% | <0.0001 | 72 | Fig 4A |
|  |  |  |  |  |  |
| N2 unmated | 10.6±0.3 | -- | -- | 68 | Fig 4-S1L |
| N2 Day 3 2h mated with *fog-2* males | 10.8±0.3 | +2% | 0.3575 | 70 | Fig 4-S1L |
|  |  |  |  |  |  |
| **Experiment 26** |  |  |  |  |  |
| *hlh-30(tm1978)* unmated | 11.7±0.3 | -- | -- | 74 |  |
| *hlh-30(tm1978)* Day 3 2h mated with *fog-2* males | 9.8±0.3 | -16% | 0.0002 | 75 |  |
|  |  |  |  |  |  |
| N2 unmated | 13.4±0.5 | -- | -- | 61 |  |
| N2 Day 3 2h mated with *fog-2* males | 12.7±0.4 | -5% | 0.2263 | 76 |  |
|  |  |  |  |  |  |
| **Experiment 27** |  |  |  |  |  |
| N2 unmated | 13.2±0.5 | -- | -- | 58 | Fig 4J |
| N2 Day 3 12h mated with *fog-2* males | 11.0±0.3 | -17% | 0.0005 | 70 | Fig 4J |
| N2 Day 3 24h mated with *fog-2* males | 9.0±0.4 | -32% | <0.0001 | 63 | Fig 4J |
|  |  |  |  |  |  |
| HLH-30::GFP(BC11288) unmated | 11.9±0.4 | -- | -- | 81 | Fig 4K |
| HLH-30::GFP(BC11288) Day 3 12h mated with *fog-2* males | 11.6±0.4 | -3% | 0.4471 | 53 | Fig 4K |
| HLH-30::GFP(BC11288) Day 3 24h mated with *fog-2* males | 9.7±0.4 | -18% | 0.0001 | 61 | Fig 4K |
|  |  |  |  |  |  |
| **Experiment 28** |  |  |  |  |  |
| N2 unmated | 15.6±0.7 | -- | -- | 52 |  |
| N2 Day 3 12h mated with *fog-2* males | 12.4±0.5 | -20% | 0.0001 | 44 |  |
| N2 Day 3 24h mated with *fog-2* males | 11.8±0.4 | -24% | <0.0001 | 55 |  |
|  |  |  |  |  |  |
| HLH-30::GFP(BC11288) unmated | 14.5±0.3 | -- | -- | 68 |  |
| HLH-30::GFP(BC11288) Day 3 12h mated with *fog-2* males | 14.7±0.4 | 1% | 0.6558 | 53 |  |
| HLH-30::GFP(BC11288) Day 3 24h mated with *fog-2* males | 11.7±0.6 | -19% | <0.0001 | 48 |  |
|  |  |  |  |  |  |
| **Experiment 29** |  |  |  |  |  |
| *fog-2(q71)* on *let-363* RNAi unmated | 13.3±0.3 | -- | -- | 62 | Fig 5C |
| *fog-2(q71)* on *let-363* RNAi Day 3 2h mated with *fog-2* males | 13.2±0.4 | -1% | 0.7368 | 72 | Fig 5C |
|  |  |  |  |  |  |
| *fog-2(q71)* on ctrl RNAi unmated | 18.0±0.7 | -- | -- | 57 | Fig 5D |
| *fog-2(q71)* on ctrl RNAi Day 3 2h mated with *fog-2* males | 12.4±0.5 | -31% | <0.0001 | 71 | Fig 5D |
|  |  |  |  |  |  |
| **Experiment 30** |  |  |  |  |  |
| *fog-2(q71)* on ctrl RNAi unmated | 17.4±0.8 | -- | -- | 31 | Fig 5-S1C |
| *fog-2(q71)* on ctrl RNAi Day 3 2h mated with *fog-2* males | 11.7±0.6 | -33% | <0.0001 | 33 | Fig 5-S1C |
|  |  |  |  |  |  |
| *fog-2(q71)* on *rict-1* RNAi unmated | 15.2±0.6 | -- | -- | 50 | Fig 5-S1D |
| *fog-2(q71)* on *rict-1* RNAi Day 3 2h mated with *fog-2* males | 14.9±0.6 | -2% | 0.8375 | 60 | Fig 5-S1D |
|  |  |  |  |  |  |
| *fog-2(q71)* on *daf-15* RNAi unmated | 15.9±0.6 | -- | -- | 52 | Fig 5-S1E |
| *fog-2(q71)* on *daf-15* RNAi Day 3 2h mated with *fog-2* males | 15.2±0.6 | -4% | 0.6903 | 58 | Fig 5-S1E |
|  |  |  |  |  |  |
| *fog-2(q71)* on *raga-1* RNAi unmated | 15.3±0.5 | -- | -- | 60 | Fig 5E |
| *fog-2(q71)* on *raga-1* RNAi Day 3 2h mated with *fog-2* males | 14.3±0.6 | -6% | 0.2378 | 60 | Fig 5E |
|  |  |  |  |  |  |
| *raga-1(ok701)* unmated | 18.5±0.7 | -- | -- | 49 | Fig 5F |
| *raga-1(ok701)* Day 7 2h mated with *fog-2* males | 18.1±0.7 | -2% | 0.5833 | 50 | Fig 5F |
|  |  |  |  |  |  |
| **Experiment 31** |  |  |  |  |  |
| *hlh-30(tm1978)* on *let-363* RNAi unmated | 13.8±0.3 | -- | -- | 75 | Fig 5G |
| *hlh-30(tm1978)* on *let-363* RNAi Day 3 2h mated with *fog-2* males | 10.5±0.4 | -34% | <0.0001 | 64 | Fig 5G |
|  |  |  |  |  |  |
| *hlh-30(tm1978)* on ctrl RNAi unmated | 10.4±0.2 | -- | -- | 74 | Fig 5-S1H |
| *hlh-30(tm1978)* on ctrl RNAi Day 3 2h mated with *fog-2* males | 9.2±0.2 | -12% | <0.0001 | 63 | Fig 5-S1H |
|  |  |  |  |  |  |
| *fog-2(q71)* on *let-363* RNAi unmated | 13.0±0.4 | -- | -- | 50 | Fig 5-S1G |
| *fog-2(q71)* on *let-363* RNAi Day 3 2h mated with *fog-2* males | 12.9±0.4 | -1% | 0.8275 | 66 | Fig 5-S1G |
|  |  |  |  |  |  |
| *fog-2(q71)* on ctrl RNAi unmated | 13.7±0.6 | -- | -- | 53 |  |
| *fog-2(q71)* on ctrl RNAi Day 3 2h mated with *fog-2* males | 10.1±0.4 | -26% | <0.0001 | 58 |  |
|  |  |  |  |  |  |
| **Experiment 32** |  |  |  |  |  |
| N2 on l4440 control RNAi unmated | 14.9±0.3 | -- | -- | 55 | Fig 5-S1A |
| N2 on l4440 control RNAi Day 3 2h mated with *fog-2* males | 14.9±0.4 | 0% | 0.7888 | 49 | Fig 5-S1A |
|  |  |  |  |  |  |
| N2 on *let-363* RNAi unmated | 12.3±0.5 | -- | -- | 52 | Fig 5-S1B |
| N2 on *let-363* RNAi Day 3 2h mated with *fog-2* males | 12.1±0.6 | -2% | 0.8791 | 54 | Fig 5-S1B |
|  |  |  |  |  |  |
| **Experiment 33** |  |  |  |  |  |
| N2 on l4440 control RNAi unmated | 13.0±0.4 | -- | -- | 50 |  |
| N2 on l4440 control RNAi Day 3 2h mated with *fog-2* males | 12.8±0.4 | -2% | 0.8824 | 50 |  |
|  |  |  |  |  |  |
| N2 on *let-363* RNAi unmated | 14.4±0.5 | -- | -- | 42 |  |
| N2 on *let-363* RNAi Day 3 2h mated with *fog-2* males | 14.0±0.6 | -3% | 0.7068 | 47 |  |
|  |  |  |  |  |  |
| *hlh-30(tm1978)* on *let-363* RNAi unmated | 14.9±0.3 | -- | -- | 62 |  |
| *hlh-30(tm1978)* on *let-363* RNAi Day 3 2h mated with *fog-2* males | 11.6±0.4 | -22% | 0.0001 | 37 |  |
|  |  |  |  |  |  |
| **Experiment 34** |  |  |  |  |  |
| *fog-2(q71)* on *let-363* RNAi unmated | 13.3±0.3 | -- | -- | 68 |  |
| *fog-2(q71)* on *let-363* RNAi Day 3 2h mated with *fog-2* males | 12.9±0.3 | -3% | 0.4919 | 66 |  |
|  |  |  |  |  |  |
| *fog-2(q71)* on ctrl RNAi unmated | 16.3±0.7 | -- | -- | 58 |  |
| *fog-2(q71)* on ctrl RNAi Day 3 2h mated with *fog-2* males | 13.4±0.5 | -18% | 0.0010 | 61 |  |
|  |  |  |  |  |  |
| **Experiment 35** |  |  |  |  |  |
| *pqm-1(ok485)* unmated | 12.5±0.5 | -- | -- | 50 | Fig 6B |
| *pqm-1(ok485)* 24h mated with *fog-2* males | 12.5±0.5 | 0% | 0.8407 | 50 | Fig 6B |
|  |  |  |  |  |  |
| N2 unmated | 10.6±0.4 | -- | -- | 50 | Fig 6-S1A |
| N2 24h mated with *fog-2* males | 8.7±0.4 | -18% | 0.0127 | 50 | Fig 6-S1A |
|  |  |  |  |  |  |
| **Experiment 36** |  |  |  |  |  |
| *pqm-1(ok485)* unmated | 11.0±0.2 | -- | -- | 66 |  |
| *pqm-1(ok485)* Day 3 2h mated with *fog-2* males | 11.0±0.3 | 0% | 0.8895 | 50 |  |
|  |  |  |  |  |  |
| N2 unmated | 12.7±0.4 | -- | -- | 60 |  |
| N2 Day 3 2h mated with *fog-2* males | 13.3±0.6 | 5% | 0.2742 | 55 |  |
|  |  |  |  |  |  |
| **Experiment 37** |  |  |  |  |  |
| *pqm-1(ok485)* on ctrl RNAi unmated | 10.5±0.3 | -- | -- | 67 | Fig 6A |
| *pqm-1(ok485)* on ctrl RNAi Day 3 2h mated with *fog-2* males | 11.1±0.4 | +6% | 0.2070 | 63 | Fig 6A |
|  |  |  |  |  |  |
| *pqm-1(ok485)* on *hlh-30* RNAi unmated | 11.6±0.2 | -- | -- | 77 |  |
| *pqm-1(ok485)* on *hlh-30* RNAi Day 3 2h mated with *fog-2* males | 11.0±0.3 | -5% | 0.0739 | 66 |  |
|  |  |  |  |  |  |
| *hlh-30(tm1978)* on *pqm-1* RNAi unmated | 11.1±0.2 | -- | -- | 72 |  |
| *hlh-30(tm1978)* on *pqm-1* RNAi Day 3 2h mated with *fog-2* males | 10.9±0.2 | -2% | 0.2984 | 63 |  |
|  |  |  |  |  |  |
| *hlh-30(tm1978)* on ctrl RNAi unmated | 12.6±0.3 | -- | -- | 63 |  |
| *hlh-30(tm1978)* on ctrl RNAi Day 3 2h mated with *fog-2* males | 11.5±0.3 | -9% | 0.0020 | 63 |  |
|  |  |  |  |  |  |
| **Experiment 38** |  |  |  |  |  |
| *pqm-1(ok485)* on ctrl RNAi unmated | 13.2±0.4 | -- | -- | 64 | Fig 6G |
| *pqm-1(ok485)* on ctrl RNAi Day 3 2h mated with *fog-2* males | 13.1±0.5 | -1% | 0.4169 | 76 | Fig 6G |
|  |  |  |  |  |  |
| *pqm-1(ok485)* on *hlh-30* RNAi unmated | 13.2±0.3 | -- | -- | 71 | Fig 6G |
| *pqm-1(ok485)* on *hlh-30* RNAi Day 3 2h mated with *fog-2* males | 13.1±0.3 | -1% | 0.9161 | 71 | Fig 6G |
|  |  |  |  |  |  |
| *hlh-30(tm1978)* on ctrl RNAi unmated | 13.1±0.4 | -- | -- | 62 | Fig 6-S1C |
| *hlh-30(tm1978)* on ctrl RNAi Day 3 2h mated with *fog-2* males | 11.6±0.3 | -11% | 0.0015 | 68 | Fig 6-S1C |
|  |  |  |  |  |  |
| *hlh-30(tm1978)* on *pqm-1* RNAi unmated | 11.7±0.2 | -- | -- | 66 | Fig 6-S1C |
| *hlh-30(tm1978)* on *pqm-1* RNAi Day 3 2h mated with *fog-2* males | 12.0±0.2 | +3% | 0.3601 | 74 | Fig 6-S1C |
|  |  |  |  |  |  |
| **Experiment 39** |  |  |  |  |  |
| *fog-2(q71)* on ctrl RNAi unmated | 18.4±0.9 | -- | -- | 54 | Fig 7E |
| *fog-2(q71)* on ctrl RNAi Day 3 2h mated with *fog-2* males | 13.3±0.7 | -28% | <0.0001 | 51 | Fig 7E |
| *fog-2(q71)* on ctrl RNAi Day 3 2h mated with *fer-6* males | 15.4±0.6 | -16% | 0.0032 | 49 | Fig 7E |
|  |  |  |  |  |  |
| *fog-2(q71)* on *ins-8* RNAi unmated | 18.4±0.7 | -- | -- | 53 | Fig 7C |
| *fog-2(q71)* on *ins-8* RNAi Day 3 2h mated with *fog-2* males | 17.2±0.9 | -7% | 0.2879 | 46 | Fig 7C |
| *fog-2(q71)* on *ins-8* RNAi Day 3 2h mated with *fer-6* males | 17.3±0.7 | -6% | 0.2582 | 47 | Fig 7C |
|  |  |  |  |  |  |
| *fog-2(q71)* on *ins-7* RNAi unmated | 18.2±0.6 | -- | -- | 47 | Fig 7D |
| *fog-2(q71)* on *ins-7* RNAi Day 3 2h mated with *fog-2* males | 17.7±1.1 | -3% | 0.9380 | 45 | Fig 7D |
| *fog-2(q71)* on *ins-7* RNAi Day 3 2h mated with *fer-6* males | 17.2±0.7 | -5% | 0.3994 | 47 | Fig 7D |
|  |  |  |  |  |  |
| *fog-2(q71)* on *let-363* RNAi unmated | 16..0±0.5 | -- | -- | 56 | Fig 5-S1F |
| *fog-2(q71)* on *let-363* RNAi Day 3 2h mated with *fer-6* males | 16.0±0.6 | 0% | 0.8488 | 48 | Fig 5-S1F |
|  |  |  |  |  |  |
| **Experiment 40** |  |  |  |  |  |
| *fog-2(q71)* on ctrl RNAi unmated | 17.4±0.8 | -- | -- | 31 |  |
| *fog-2(q71)* on ctrl RNAi Day 3 2h mated with *fog-2* males | 11.7±0.6 | -33% | <0.0001 | 33 |  |
| *fog-2(q71)* on ctrl RNAi Day 3 2h mated with *fer-6* males | 13.8±0.6 | -21% | 0.0007 | 34 |  |
|  |  |  |  |  |  |
| *fog-2(q71)* on *ins-8* RNAi unmated | 13.9±0.7 | -- | -- | 43 |  |
| *fog-2(q71)* on *ins-8* RNAi Day 3 2h mated with *fog-2* males | 14.2±0.7 | 2% | 0.6984 | 47 |  |
| *fog-2(q71)* on *ins-8* RNAi Day 3 2h mated with *fer-6* males | 13.9±0.7 | 0% | 0.7111 | 45 |  |
|  |  |  |  |  |  |
| *fog-2(q71)* on *ins-7* RNAi unmated | 13.7±0.5 | -- | -- | 48 |  |
| *fog-2(q71)* on *ins-7* RNAi Day 3 2h mated with *fog-2* males | 15.0±0.5 | 9% | 0.0124 | 60 |  |
| *fog-2(q71)* on *ins-7* RNAi Day 3 2h mated with *fer-6* males | 13.6±0.6 | -1% | 0.4689 | 54 |  |
|  |  |  |  |  |  |
| *fog-2(q71)* on *let-363* RNAi unmated | 11.2±0.4 | -- | -- | 49 |  |
| *fog-2(q71)* on *let-363* RNAi Day 3 2h mated with *fog-2* males | 10.5±0.4 | -6% | 0.3783 | 53 |  |
| *fog-2(q71)* on *let-363* RNAi Day 3 2h mated with *fer-6* males | 12.6±0.4 | 12% | 0.0280 | 54 |  |
|  |  |  |  |  |  |
